# Specific Physical Exercise Improves Energetic Metabolism in the Skeletal Muscle of Amyotrophic-Lateral- Sclerosis Mice

**DOI:** 10.3389/fnmol.2017.00332

**Published:** 2017-10-20

**Authors:** Céline Desseille, Séverine Deforges, Olivier Biondi, Léo Houdebine, Domenico D’amico, Antonin Lamazière, Cédric Caradeuc, Gildas Bertho, Gaëlle Bruneteau, Laure Weill, Jean Bastin, Fatima Djouadi, François Salachas, Philippe Lopes, Christophe Chanoine, Charbel Massaad, Frédéric Charbonnier

**Affiliations:** ^1^Sorbonne Paris Cité, Faculté des Sciences Fondamentales et Biomédicales, Université Paris Descartes, Paris, France; ^2^INSERM, UMR-S 1124, Paris, France; ^3^Laboratoire de lipidomique, Faculté de Médecine Pierre et Marie Curie - Hôpital Saint-Antoine, Université Paris 6, Paris, France; ^4^UMR 8601 CNRS, Université Paris Descartes, Paris, France; ^5^Hôpital de la Salpêtrière, Département des Maladies du Système Nerveux, Equipe Neurogénétique et Physiologie, Institut du Cerveau et de la Moelle, Paris, France; ^6^UFR Sciences Fondamentales Appliquées, Département STAPS, Université d’Evry-Val-d’Essonne, Evry, France

**Keywords:** amyotrophic lateral sclerosis, swimming, running, lipid, glucose metabolism, autophagy, fat deposition

## Abstract

Amyotrophic Lateral Sclerosis is an adult-onset neurodegenerative disease characterized by the specific loss of motor neurons, leading to muscle paralysis and death. Although the cellular mechanisms underlying amyotrophic lateral sclerosis (ALS)-induced toxicity for motor neurons remain poorly understood, growing evidence suggest a defective energetic metabolism in skeletal muscles participating in ALS-induced motor neuron death ultimately destabilizing neuromuscular junctions. In the present study, we report that a specific exercise paradigm, based on a high intensity and amplitude swimming exercise, significantly improves glucose metabolism in ALS mice. Using physiological tests and a biophysics approach based on nuclear magnetic resonance (NMR), we unexpectedly found that SOD1(G93A) ALS mice suffered from severe glucose intolerance, which was counteracted by high intensity swimming but not moderate intensity running exercise. Furthermore, swimming exercise restored the highly ALS-sensitive *tibialis* muscle through an autophagy-linked mechanism involving the expression of key glucose transporters and metabolic enzymes, including GLUT4 and glyceraldehyde-3-phosphate dehydrogenase (GAPDH). Importantly, GLUT4 and GAPDH expression defects were also found in muscles from ALS patients. Moreover, we report that swimming exercise induced a triglyceride accumulation in ALS *tibialis*, likely resulting from an increase in the expression levels of lipid transporters and biosynthesis enzymes, notably DGAT1 and related proteins. All these data provide the first molecular basis for the differential effects of specific exercise type and intensity in ALS, calling for the use of physical exercise as an appropriate intervention to alleviate symptoms in this debilitating disease.

## Introduction

Amyotrophic lateral sclerosis (ALS), the most widespread adult-onset motor neuron disease, is a chronic neurodegenerative affection characterized by a progressive motor weakness originating from the selective loss of motor neurons. Death typically occurs within 3 years of onset, usually from respiratory failure (Al-Chalabi and Hardiman, 2013). Riluzole, the only currently available drug extends survival by approximately 3 months (Lacomblez et al., 1996), pointing out crying need for the development of new therapeutic strategies. In approximately 85–90% of cases, ALS is sporadic (sALS). The 10–15% of remaining cases, referred to as familial ALS (fALS), are characterized by the frequent (20% of the cases) occurrence of mutations in superoxide dismutase 1 (*SOD1*) gene (Rosen et al., 1993), leading to the development of mouse models of ALS (Gurney et al., 1994). The clinical and morphological abnormalities are identical in sALS and fALS, suggesting the disruption of similar mechanisms. Although the precise identity of ALS-triggered degeneration mechanisms is still elusive, growing evidence points toward alterations in metabolic status (Desport et al., 2001; Bouteloup et al., 2009; Pradat et al., 2010; Dupuis et al., 2011; Lindauer et al., 2013) thought to mainly derive from skeletal muscle lipid hypermetabolism (Poulton and Rossi, 1993; Pradat et al., 2010; Dupuis et al., 2011). This feature results in a frequent, severe and progressive weight loss, which represents an independent negative prognostic factor for survival in ALS (Desport et al., 1999; Dupuis et al., 2011; Paganoni et al., 2011). Accordingly, ALS risk and progression decrease in individuals with high body mass index and type 2 diabetes mellitus (Kioumourtzoglou et al., 2015; Mariosa et al., 2015). These data suggest that the carbohydrate metabolism could be also intrinsically altered in ALS, concurring with the significant number of ALS patients suffering from glucose intolerance (Dupuis et al., 2011; Paganoni et al., 2011). However, the data obtained in different ALS mouse models are inconsistent. Yet, no defect in glucose tolerance was found in SOD1(G93A) ALS mice (Smittkamp et al., 2014) while an excessive glucose uptake was recorded in SOD1(G86R) ALS mice (Dupuis et al., 2004b). In pre-symptomatic SOD1(G86R) ALS mice, despite more glucose consumption, a metabolic switch toward lipid use was found in fast-twitch muscles, possibly due to the inhibition of pyruvate dehydrogenase (PDH), a pivotal enzyme of carbohydrate catabolism (Kioumourtzoglou et al., 2015).

In the myofibers, as in other cells, autophagy can play a major role in the energetic metabolism balance in case of starvation or huge energetic demand. It provides energy by degrading cellular intrinsic components to produce the three main energetic macromolecules, i.e., glucose, lipids, and amino acids (Singh and Cuervo, 2011). Importantly, autophagy was found altered in ALS muscles, with in particular, abnormal expressions of several autophagic markers, including LC3B and P62, depicted in the skeletal muscle of ALS mice (Dobrowolny et al., 2008; Crippa et al., 2013) or ALS-mimicking muscle cell cultures (Onesto et al., 2011). In addition, it has been recently reported that the autophagic activity is compromised in the skeletal muscle of ALS mice (Xiao et al., 2015). These perturbations occur in ALS muscles although aggregates of mSOD1 are unexpected (Wei et al., 2012), suggesting that, in ALS muscles, it is the macro-autophagy, involved in energy supply that is affected, and not the micro-autophagy, involved in the elimination of protein aggregates. Despite all these observations, a functional link between autophagy and lipid and carbohydrate metabolisms in ALS muscles is still lacking. Thus, although further investigations are required to draw a picture of the metabolic defects occurring in ALS, all these initial results suggest that modulating skeletal muscle energetic metabolism might constitute a promising avenue for alleviating ALS symptoms.

In this context, specific physical exercises are expected to differentially shift the muscular energy metabolism either toward an oxidative pattern, notably lipidic, in case of low-intensity exercise, or toward a glycolytic metabolism, in case of high-intensity exercise (Romijn et al., 1993; van Loon et al., 2001). Furthermore, important metabolic effects of physical exercise have been linked to the activity-dependent induction of autophagy in skeletal muscle (Jaspers et al., 2017). These effects include the adaptation of muscle glucose homeostasis and the protection against glucose intolerance (He et al., 2012) and the protection of mitochondria against exercise-induced damages. Interestingly, submitting SOD1(G93A) ALS mice to a swimming-based protocol, involving a high intensity and amplitude exercise (Grondard et al., 2008), resulted in a remarkable increase in lifespan and neuroprotection at the lumbar motor neurons level (Deforges et al., 2009). By contrast, in most of the available studies, submitting mSOD1 ALS mice to different running-based programs conferred a reduced neuroprotection (Kirkinezos et al., 2003; Mahoney et al., 2004; Kaspar et al., 2005; Deforges et al., 2009; Carreras et al., 2010). Data in humans are even more confusing, and the use of exercise in ALS patients remains highly debated (Harwood et al., 2009; Pupillo et al., 2014). Although these controversies could result from the different characteristics of exercise and the heterogeneity of protocols used, the exercise-induced impact on energetic metabolism and on autophagy in ALS skeletal muscle has never been investigated.

In the present study, using human ALS muscle biopsies and SOD1(G93A) ALS mice, we report that the carbohydrate catabolism is impaired in ALS, and that physical exercise, if adequately designed, can reduce muscle metabolic defects likely through the improvement of skeletal muscle autophagy.

## Materials and Methods

### Human Biopsies

Human muscle biopsies were obtained from 10 ALS patients referred from the Paris Motor Neuron Disease Center (Pitié-Salpêtrière Hospital, Paris, France). All patients met the El Escorial World Federation of Neurology criteria for the diagnosis of definite, probable or possible ALS (Brooks et al., 2000). Muscle specimens were taken from the anconeus muscle for three patients and the deltoid muscle for seven patients. Six subjects with histochemically normal deltoid muscle were used as controls. These subjects had undergone muscle biopsy as a diagnostic procedure and, after careful clinical and histological evaluation, had been considered free of any neuromuscular disorder. The study was approved by the local ethics committee, and all patients provided written informed consent before muscle biopsy, according to institutional guidelines. Biopsy specimens were immediately frozen in liquid nitrogen, and stored at -80°C until use.

### Ethics Statement

The care and treatment of animals followed the French national authority guidelines for the detention, use and the ethical treatment of laboratory animals. All experimental procedures that include minimizing the number of animals used and their suffering were approved by the policies of the French Agriculture and Forestry Ministry. All the experiments using mice were performed in a blind systematic fashion so as to minimize bias.

### Mice, Exercise and Treatments

Transgenic male B6/SJL mice with the G93A human SOD1 mutation [B6/SJL-Tg (SOD1-G93A) 1Gur/J] (ALS mice) were purchased from The Jackson Laboratory (Bar Harbor, ME, United States). ALS male were crossed with wild-type B6/SJL females (Janvier, Le Genest-Saint-Isle, France), and only males were used for this study.

Amyotrophic lateral sclerosis males were trained from 70 days of age (P70), 30 min a day, 5 days a week, to a running-based or swimming-based exercise as previously described (Grondard et al., 2008; Deforges et al., 2009) and until 115 days of age (P115). 18 ALS mice (Run ALS) were submitted to a running-based training on a speed-regulated treadmill (max 13 m.min^-1^). Eighteen ALS mice (Swim ALS) were submitted to a swimming-based training in an adjustable-flow swimming pool (max 5 L.min^-1^). These groups were compared to 29 Sedentary ALS and 20 Sedentary Control mice.

For the autophagy analysis, specific groups of ALS males at P70 were subjected to the same swimming-based exercise for 3 days, and treated for autophagy inhibition as previously reported (Kang et al., 2014). Accordingly, each mice was intraperitoneally injected 1 h before exercise with 10 μl.g^-1^ of either chloroquine (CQ) at 5 μg.μl^-1^, or 3-methyladenine (3-MA) at 1.5 μg.μl^-1^, or a placebo NaCl 0.9% solution.

### Glucose Tolerance Test and Lactate Measurement

Glucose tolerance tests were performed on 4 or 12 h fasted mice at P70 and P115, with the same results. Mice were intraperitoneally injected with D-glucose (Sigma–Aldrich, St. Louis, MO, United States) at a dose of 2 g.kg^-1^. Glucose concentration was then measured every 15 min using a Glucotrend R analyser (Boehringer Mannheim GmbH, Germany) from whole blood samples taken from the tail.

Blood lactate samples were collected from right ventricle on anesthetized mice, with 1% pentobarbital solution (6 μl.g^-1^) diluted in 0.9% saline buffer, using a 1 ml syringe mounted with a 22-gauge needle and coated with heparin (5000 UI.ml^-1^, PanPharma Luitré, France), 2 h after training. Blood samples were centrifuged (1000 *g*, 10min, +4°C) and serum was frozen at -80°C. The serum lactate level was measured using Lactate assay kit (Biovision research, Mountain view, CA, United States) following manufacturer’s instructions. For each mouse, 1 μl of serum were incubated at +22°C in 50 μl of incubation buffer and 50 μl of reaction mix in a 96-well plate, and read on spectrophotometer at 570 nm wavelength after 30 min of incubation. Lactate levels were given nmol.μl^-1^.

### Nuclear Magnetic Resonance (NMR) Analysis

Sample preparation was made using the classic protocol of tissue extraction (Beckonert et al., 2007). The combined extraction of polar and lipophilic metabolites from tissues was obtained using the methanol/chloroform/water mixture.

Muscle mass ranged between 15 and 40 mg. The 13 polar sample extracts were transferred in 5 mm nuclear magnetic resonance (NMR) tubes to obtain a final volume of 580 μL/samples.

#### Nuclear Magnetic Resonance

1H NMR spectra of aqueous tissues extract were measured at 300 K on a Bruker Avance II 500MHz spectrometer. The spectral acquisition was based on CPMG-presat (cpmgpr) a 1D CPMG pulse sequence with pre-saturation for water suppression. Parameters used for the pulse sequence were a relaxation delay of 1 s; a mixing time of 100 ms; an acquisition time of 1.36 s; a 90° pulse length of 8 μs. 32K data points were collected during 512 scans with a spectral width of 20 ppm.

#### Data Preprocessing

The signal preprocessing of the NMR spectra was performed with MestReNova 8.0 software including the following standard steps: line-broadening factor (0.3 Hz), Fourier transformation, phasing, baseline correction, calibration by the TSP, exclusion of uninteresting or noisy signals (water from 4.53 to 4.97 ppm and extremities < 0.21 ppm and >9.45 ppm), equidistant bucketing (bin width of 0.04 ppm) and constant integral normalization. Thus, we obtained a matrix of an NMR spectra dataset of 13 subjects and 224 buckets.

### RT-qPCR Analysis

Mouse tissues were collected 2 h after training in liquid nitrogen and RNA was extracted using TRizol reagent (Invitrogen, Life Technologies, Saint-Aubin, France). 1 μg of human or mouse RNAs was reverse transcribed with oligodT using reverse transcriptase Improm II (Promega France, Charbonnières, France). Quantitative real time PCR was performed with standard protocols using SYRB Green ROX detector in ABIPrism 7000 (ABgene, Courtaboeuf, France). Specific primers were used at 300 nM (**Table 1**). The relative amounts of cDNA in each sample were determined on the basis of the threshold cycle (*C*t) for each PCR product and normalized to either *RPL13* or *Rps26* Ct for human or mouse samples, respectively. These housekeeping genes has been determined as best internal controls using Bestkeeper (Pfaffl et al., 2004) and Normfinder (Andersen et al., 2004) algorithms on 10 control and 10 ALS *tibialis* and over 10 different housekeeping genes (data not shown). The analysis was done relative to respective control samples and given by 2^-ΔΔ*C*_T_^.

**Table 1:** RT-qPCR primer sequences.

Gene	GenBank ID	Forward Sequence (5′–3′)	Reverse sequence (5′–3′)	Species
GAPDH	NM_002046	ACCCAGAAGACTGTGGATGG	TCTAGACGGCAGGTCAGGTC	Human
SLC2A4 (GLUT4)	NM_001042	CTTCATCATTGGCATGGGTTT	AGGACCGCAAATAGAAGGAAGA	Human
PDK4	NM_002612	TCCACTGCACCAACGCCT	TGGCAAGCCGTAACCAAAA	Human
RPL13A	NM_012423	AAGGTCGTGCGTCTGAAG	GAGTCCGTGGGTCTTGAG	Human
Rps26	NM_013765	AGGAGAAACAACGGTCGTGCCAAA	GCGCAAGCAGGTCTGAATCGTG	Mouse
ACC	NM_133360	GCCTCTTCCTGACAAACGAG	TGACTGCCGAAACATCTCTG	Mouse
Bcl2	NM_177410	TGAACCGGCATCTGCACAC	CGTCTTCAGAGACAGCCAGGAG	Mouse
Becn1	NM_019584	TTCAAGATCCTGGACCGGGTCAC	AGACACCATCCTGGCGAGTTTC	Mouse
Cd36	NM_007643	ATTAATGGCACAGACGCAGC	TTCAGATCCGAACACAGCGT	Mouse
Dgat1	NM_010046	CCTCAGCCTTCTTCCATGAG	ACTGGGGCATCGTAGTTGAG	Mouse
Fasn	NM_007988	AGAGATCCCGAGACGCTTCT	GCCTGGTAGGCATTCTGTAGT	Mouse
Gapdh	NM_008084	GTGGACCTCATGGCCTACAT	TGT GAG GGA GAT GCT CAG TG	Mouse
Slc2a4 (Glut4)	NM_009204	CTGCAAAGCGTAGGTACCAA	CCT CCC GCC CTT AGT TG	Mouse
Map1lc3b (Lc3b)	NM_026160	CATGCCGTCCGAGAAGACCT	GATGAGCCGGACATCTTCCACT	Mouse
Sqstm1 (P62)	NM_011018	AGGGAACACAGCAAGCT	GCC AAA GTG TCC ATG TTT CA	Mouse
Pdk4	NM_013743	TGTGATGTGGTAGCAGTAGTC	ATGTGGTGAAGGTGTGAAG	Mouse
Srebf1c	NM_011480	GGAGCCATGGATTGCACATT	GGCCCGGGAAGTCACTGT	Mouse
Ucp3	NM_009464	ATGAGTTTTGCCTCCATTCG	GGCGTATCATGGCTTGAAAT	Mouse
Vldlr	NM_013703	GAGCCCCTGAAGGAATGCC	CCTATAACTAGGTCTTTGCAGATATGG	Mouse


### Immunohistochemistry

Muscles from P115 mice were frozen in cold isopentane (-80°C) and transversally cut in 10 μm sections using cryostat (CM 3050S, Leica, Le Pecq France). After an incubation in a blocking solution [Tris Buffer Saline (TBS), 0.1% tween, 0.1% triton and 4% donkey serum] for 30 min at room temperature, sections were incubated with primary polyclonal rabbit anti-GLUT4 antibody (1:50; GT41-A Alpha Diagnostic, San Antonio, TX, United States), overnight at +4°C. Sections were then washed and incubated with secondary polyclonal goat anti-rabbit Cy3-conjugated antibody (1:400; 111-165-144, Jackson laboratories, Baltimore, MD, United States) for 2 h at room temperature. Sections were washed and mounted in VECTASHIELD^®^ mounting medium (Vector Laboratories, Burlingame, CA, United States). The staining specificity was checked in control incubations performed in the absence of the primary antibody.

### Microscopy and Images Analysis

Epifluorescence was detected with a CMOS camera (ORCA Flash 2.8, Hamamatsu Photonics France, Massy, France) and histological images were recorded with a RGB camera (ICC1 Zeiss, Carl Zeiss SAS, Le Pecq, France) mounted on Zeiss AxioObserver (Z1) using the ZEN 2012 software (Carl Zeiss SAS) with 100 (10× Zeiss Plan NeoFluar NA 0.3) and 200 (20× Zeiss EC-Plan-Apo NA 0.8) magnifications.

All images were analyzed using Image J v1.47 software (National Institutes of Health, Bethesda, MD, United States). Identical brightness, contrast, and color balance adjustments were applied to all groups. GLUT4 staining was counted in positive-fibers and characterized as full membrane-stained or not.

### Western Blots

Mouse muscles were dissected and frozen in liquid nitrogen and homogenized in 50 μl/5 mg of RIPA buffer [50 mM Tris/pH 8.3, 150 mM NaCl, 0.1% SDS, 1% NP40, 10 mM NAF, 1X Protease inhibitor (Roche, BASEL, Switzerland), 1% Phosphatase inhibitor (Sigma–Aldrich, St. Louis, MO, United States)]. Protein concentration was determined in the supernatant after centrifugation (17000 *g*, 15 min, +4°C) by Bradford protein assay (Bio-Rad, Hercules, CA, United States). Protein samples (100 μg for LC3B, 40 μg for the other proteins) were submitted to 12.5% SDS PAGE electrophoresis (1.5 M Tris pH 8.3, 12.5% Acrylamide, 0.07% Bis, 0.1% SDS, 0.05% APS, 0.06% TEMED). The separated proteins were transferred on PVDF membranes (Bio-Rad) and incubated overnight at +4°C with either rabbit anti-PDHE1α P-Ser293 (1:1000; AP1062; Merck Millipore, Darmstadt, Germany), rabbit anti-PDHE1α (1:1000; ab110334, Abcam, Cambridge, United Kingdom), rabbit anti-LC3B (1:1000; NB100-2331, Novus Biological), mouse anti-GAPDH (1:10.000; MAB374, Millipore) or mouse anti-α-tubulin (1:20.000; T6074, Sigma) diluted in blocking solution. Membranes were washed and incubated in horseradish peroxidase-conjugated secondary antibody goat anti-rabbit or goat anti-mouse (1:10.000 Jackson ImmunoResearch). Revelation was performed with Amersham^TM^ ECL^TM^ Western Blotting Analysis System (GE Healthcare, Bio-Science, Upsala, Sweden). Images were done using ImageQuant LS4000 (GE Healthcare Bio-Science, Upsala, Sweden) and quantification was performed using Image J v1.47 software.

### Lipidomic Analysis

The triacylglycerols (TAG) of the Bligh and Dyer extract (Bligh and Dyer, 1959) were prepared from homogenized muscles in water and separated on a normal phase PVA-Sil column (Polymerised Vinyl Alcohol silica (5 μ) support; L 250 mm X ID 4 mm) (YMC, Kyoto, Japan). The PVA-Sil column was fitted on a Agilent 1200 HPLC equipment coupled to the electrospray ionization (ESI) source of a triple-quadrupole mass spectrometer (API3000, AB Sciex). TAG species were quantified by integration of molecular ions (as ammonium ion adducts) from full scan mass spectra and application of standard curves that relate the responses of known amounts of reference standards to that for a single internal standard as described elsewhere (Hutchins et al., 2008).

### Statistical Analysis

All data are presented as mean and standard deviation (SD). For the glucose tolerance studies, a one-way ANOVA for repeated measures with Tukey *post hoc* analysis was performed on the data. For the other studies, a Kolmogorov–Smirnov normal distribution analysis was performed on all data followed by either a student’s *t*-test for normally distributed data or a non-parametric Kruskal–Wallis test, to verify significant differences between groups (Systat v 8.0, SPSS Inc., Chicago, IL, United States). All the data presented in this study were considered as statically different when the statistical power exceeds 95% (AnaStats.fr, France). All graphics were done with GraphPad Prism v6.01 and Adobe Illustrator CS6 v16.0.3.

## Results

### A Significant Improvement in Glucose Tolerance Is Specifically Induced by the Swimming-Based Training in ALS Mice

**FIGURE 1:**
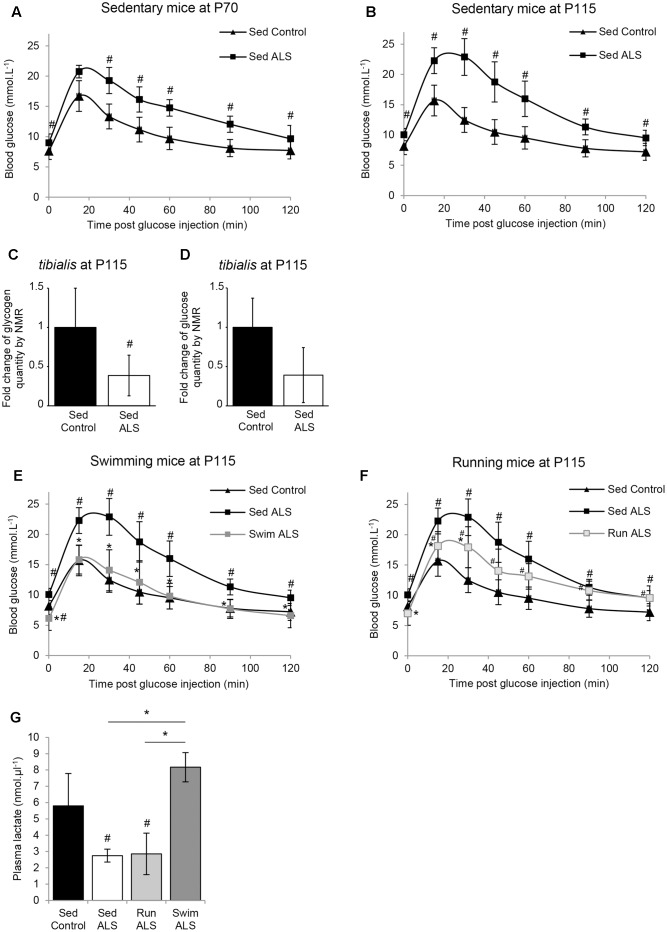
The swimming-based training improves glucose tolerance in ALS mice. **(A,B)** Glucose tolerance test in sedentary controls (Sed Control) and ALS (Sed ALS) mice at P70 **(A)** and P115 **(B)**. **(C,D)** NMR analysis of glycogen **(C)** and glucose levels **(D)** from control and ALS mouse *tibialis* muscles (control *n* = 6 and ALS mice *n* = 6). **(E,F)** Glucose tolerance test in sedentary control (Sed Control) and sedentary (Sed ALS) and trained ALS mice at P115, after a swimming- (Swim ALS) **(E)** or a running- (Run ALS) **(F)** based training (sedentary controls and ALS mice *n* = 6; trained ALS mice *n* = 4). **(G)** Blood lactate levels at P115 in Sed control, Sed ALS, and 2 h after a running (Run ALS) or a swimming session (Swim ALS) (*n* = 4). All data are shown as mean ± standard deviation (SD). # and ^∗^ indicate significance (with *P* < 0.05) relative to the control and between ALS conditions, respectively.

The ALS-induced loss of body mass detected from the asymptomatic phase of the disease in several mouse models of ALS (Dupuis et al., 2004b) is differentially altered by two different physical exercises (Deforges et al., 2009). Indeed, unlike running, the swimming-based training significantly limited the body weight loss of ALS mice (Deforges et al., 2009). Then, we questioned whether this preservation was linked to an intrinsic change in energy metabolism. To address this question, we assessed glucose tolerance in sedentary controls and sedentary and trained ALS mice in the presymptomatic (P70) and late symptomatic (P115) phases of the disease. The results of an intraperitoneal glucose tolerance test revealed that, from P70 until death, sedentary ALS mice displayed a severely impaired glucose tolerance. The blood glucose levels remained over 16 mmol.L^-1^ 1 h after a 11.1 mmol.kg^-1^ glucose injection (2 g.Kg^-1^), whereas in wild-type mice whole blood glucose concentration was closed to baseline levels at that time (**Figures 1A,B**). Since glucose intolerance was recorded for the first time in SOD1(G93A) ALS mice, we decided to verify and confirm these results by quantifying glycogen and glucose concentration levels using nuclear magnetic resonance (NMR) in the skeletal muscle of SOD1(G93A) ALS and control mice. We focused our study on the *tibialis* muscle, which is a fast-twitch muscle, mainly using glucose as fuel for energetic supply. Importantly, our NMR results revealed a significant decrease in glycogen concentration levels in ALS vs. control *tibialis* (**Figure 1C**). The concentration levels of free glucose converged toward a global decrease in the ALS *tibialis* compared to controls, although we found a high heterogeneity among samples, which is normally expected with a highly metabolized molecule (**Figure 1D**). All these data concurred with a glucose intolerance in SOD1(G93A) ALS mice and further substantiated a defect in glucose uptake.

We next recorded the effects of each training paradigm on glucose tolerance in SOD1(G93A) ALS mice. Very interestingly, the glucose tolerance in SOD1(G93A) ALS mice was significantly improved following specific training. The swimming-based trained in ALS mice completely restored the glucose tolerance at P115 (**Figure 1E**) while the running-based slightly improved it after 45 days of training (**Figure 1F**).

We next evaluated the effect of the swimming-induced increase in glucose tolerance on its subsequent use as an energetic substrate in SOD1(G93A) ALS mouse tissues. To this end, we measured the levels of circulating lactate in sedentary control and in sedentary and trained SOD1(G93A) ALS mice at P115 in resting conditions (**Figure 1G**). We found as expected (Ferraiuolo et al., 2011) a significant decrease of circulating lactate levels in sedentary SOD1(G93A) ALS mice, in comparison to control mice. Interestingly, unlike running, the swimming-based training induced a significant increase in the levels of circulating lactate in SOD1(G93A) ALS mice, suggesting a specific-exercise-induced activation of glycolysis in skeletal muscles.

All these results converge on the idea that glucose resistance in SOD1(G93A) ALS mice, also found in ALS patients, impacts muscle metabolism, as suggested by the abnormal decrease in circulating lactate. Interestingly, only the swimming-based training managed to limit this metabolic defect.

### Expression and Distribution of GLUT4 Are Altered in the ALS Skeletal Muscle and Improved by Physical Exercise

**FIGURE 2:**
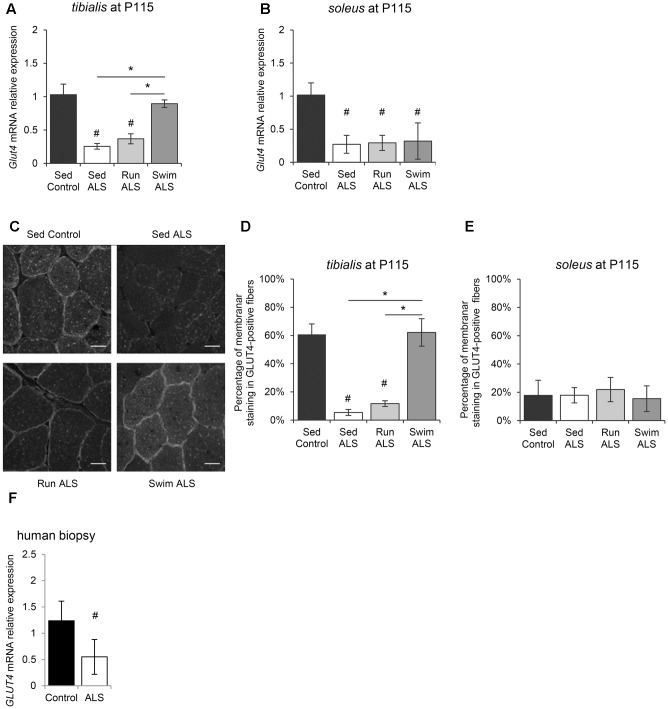
Physical exercise enhances expression and localization of GLUT4 in ALS skeletal muscles. **(A,B)** Quantification by RT-qPCR of *Glut4* mRNA expression levels in *tibialis* **(A)** and in *soleus* **(B)** of Sed Control, Sed ALS, Run ALS, and Swim ALS mice at P115. mRNA expression levels were normalized with *Rps26* mRNA**. (C–E)** Immunodetection of GLUT4 in muscle transversal section in *tibialis* **(C)** (scale bar: 20 μm) and analysis of membrane staining of GLUT4 in *tibialis* stained fibers **(D)** and *soleus* **(E)** from Sed Control, Sed ALS, Run ALS, and Swim ALS mice at P115. **(F)** Quantification by RT-qPCR of *GLUT4* mRNA expression levels in human muscular biopsies (control patients *n* = 4; ALS patients *n* = 7). mRNA expression levels were normalized with *RPL13* mRNA. All data are shown as mean ± SD. # and ^∗^ indicate significance (with *P* < 0.05) relative to the control and between ALS conditions, respectively.

We next investigated the molecular mechanisms underlying, in the one hand, glucose intolerance in SOD1(G93A) ALS mice and, on the other, specific exercise-induced potential restoration of glucose uptake by ALS mouse tissues, including skeletal muscles. Among glucose transporters, GLUT4 is the most abundant in skeletal muscle and its level of expression is a major determinant of glucose uptake by skeletal muscle fibers (Richter and Hargreaves, 2013). We analyzed GLUT4 expression in skeletal muscles of SOD1(G93A) ALS mice, particularly the fast-twitch *tibialis* and the slow-twitch *soleus*, affected earlier and later by the disease, respectively (Hegedus et al., 2008). We found a significant decrease of *Glut4* mRNA expression in the two muscles in ALS mice, in the late symptomatic phase of the disease (P115) (**Figures 2A,B**). Interestingly, physical exercise enhanced *Glut4* expression in ALS muscles, but with exercise and muscle specificities. Indeed, only the swimming-based training was able to significantly increased *Glut4* expression and specifically in the ALS *tibialis* (**Figures 2A,B**).

Since muscle glucose uptake is not only dependent on GLUT4 expression levels, but also of its abundance at the myofiber sarcolemma level, we analyzed the sub-cellular distribution of GLUT4 in the *tibialis* and *soleus* of SOD1(G93A) mice. We found that the GLUT4 subcellular distribution was largely intracellular, throughout the cytosol, and mostly absent at the sarcolemma level in ALS *tibialis* muscles, compared to controls (**Figures 2C,D**). By contrast, no difference in GLUT4 subcellular distribution was observed in the ALS *soleus* compared to controls (**Figure 2E**). Since exercise is thought to induce GLUT4 mobilization to the sarcolemma (Douen et al., 1990; Lauritzen et al., 2010), we next investigated whether, depending of its type, exercise training could restore GLUT4 subcellular distribution in the ALS muscles. In contrast to the running-based training, the swimming-based training induced a sub-cellular redistribution of the transporter in ALS *tibialis* as evidenced by both the increase in GLUT4 staining at the cell periphery and the decrease in GLUT4 staining throughout the cytoplasm of the muscle cells (**Figures 2C,D**). In the ALS *soleus*, both exercise types had no effect on GLUT4 subcellular distribution (**Figure 2E**).

Since these data substantiated a defect in glucose transport in myofibers in SOD1(G93A) ALS mice, we evaluated *GLUT4* expression in human samples from ALS patients by RT-qPCR. Consistent with ALS mouse data, we found a significant decrease in *GLUT4* mRNA expression in ALS human muscles compared to controls (**Figure 2F**).

Taken together, these results suggested an alteration of GLUT4 expression, the major glucose transporter in the skeletal muscle, specifically in the fast-twitch muscles of SOD1(G93A) ALS mice. More importantly, the GLUT4 expression was restored by the swimming-based training, suggesting a link between GLUT4 expression defects and the glucose tolerance status in sedentary and trained ALS mice.

### GAPDH Expression Is Altered in ALS Skeletal Muscles and Promoted by Physical Exercise

**FIGURE 3:**
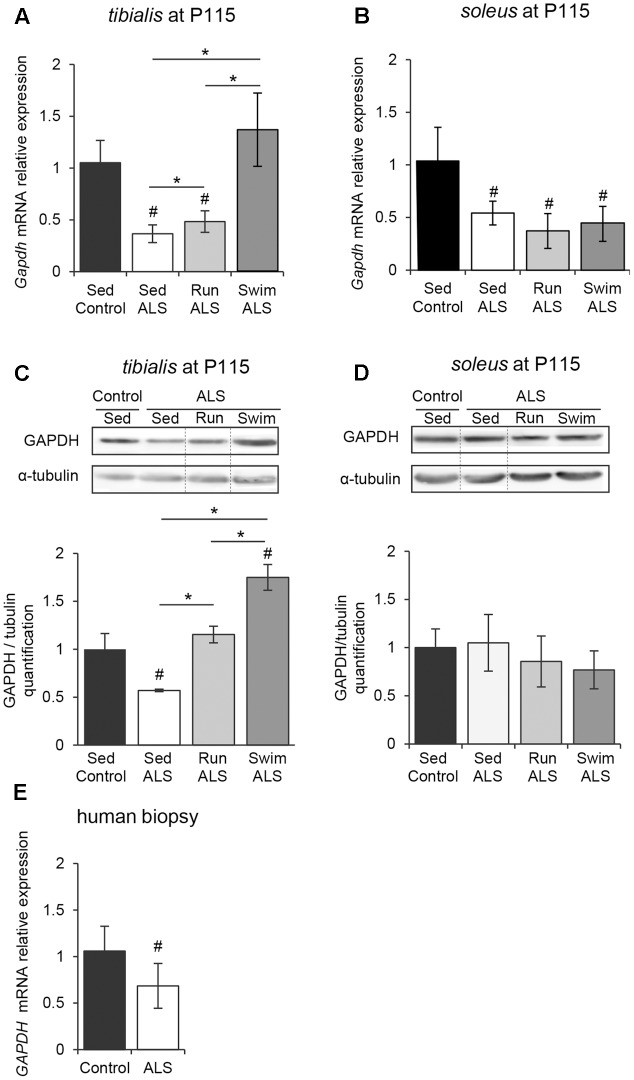
The swimming-based training enhances the GAPDH expression in ALS skeletal muscles. **(A,B)** Quantification by RT-qPCR of *Gapdh* mRNA expression levels in *tibialis* **(A)** and *soleus* **(B)** from Sed Control, Sed ALS, Run ALS, and Swim ALS mice at P115 (*n* = 5). mRNA expression levels were normalized with *Rps26* mRNA. **(C,D)** Western blot analysis (upper panel) and quantification (lower panel) of GAPDH protein in *tibialis* **(C)** and *soleus* **(D)** of Sed Control, Sed ALS, Run ALS, and Swim ALS mice at P115 (*n* = 3). Dotted lines on Western blot images symbolize some removed interspacing lanes for a side-by-side display of samples from all groups. **(E)** Quantification by RT-qPCR of *GAPDH* mRNA expression levels in human muscular biopsies (control patient *n* = 6; ALS patient *n* = 10) mRNA expression levels were normalized with *RPL13* mRNA. All data are shown as mean ± SD. # and ^∗^ indicate significance (with *P* < 0.05) relative to the control and between ALS conditions, respectively.

In order to determine if the potential defects in glucose uptake, suggested by (1) a glucose intolerance, (2) a decrease in lactate output and (3) defects in muscular GLUT4 expression, would impact glycolysis in ALS muscles, we analyzed the expression pattern of the glyceraldehyde-3-phosphate dehydrogenase (GAPDH). Indeed, GAPDH has long been recognized as a pivotal enzyme for the production of ATP and pyruvate from glucose through glycolysis. We found that, in both *tibialis* and *soleus* muscles of SOD1(G93A) ALS mice, the levels of *Gapdh* mRNA were significantly decreased in the late symptomatic phase of the disease (P115), with a more pronounced decrease in *tibialis* than in *soleus* when compared to the controls (**Figures 3A,B**). In contrast with *soleus*, we recorded a significant decrease in GAPDH protein in *tibialis* muscle consistent with mRNA expression level (**Figures 3C,D** and Supplementary Figures S1A,B).

We next investigated whether exercise training could improve GAPDH expression in ALS muscles. In the ALS *tibialis*, both exercise paradigms resulted in a significant increase in GAPDH mRNA and protein levels with the strongest efficiency observed with the swimming-based training (**Figures 3A,C**). In the ALS *soleus*, no physical training effect could be recorded on GAPDH mRNA and protein levels (**Figures 3B,D**).

Importantly, we confirmed these data in human muscle samples, where we found a significant decrease in *GAPDH* mRNA expression in ALS patients (**Figure 3E**).

Taken together, these data suggested that glycolysis is altered in ALS skeletal muscles and that high-intensity exercise could efficiently re-balanced the enzymatic equipment in ALS muscle, leading to the improvement in anaerobic capacity of fast-twitch muscles.

### The Swimming-Induced Alterations of the Metabolic Routes in ALS Skeletal Muscles Are Independent of Pyruvate Dehydrogenase Modulation

**FIGURE 4:**
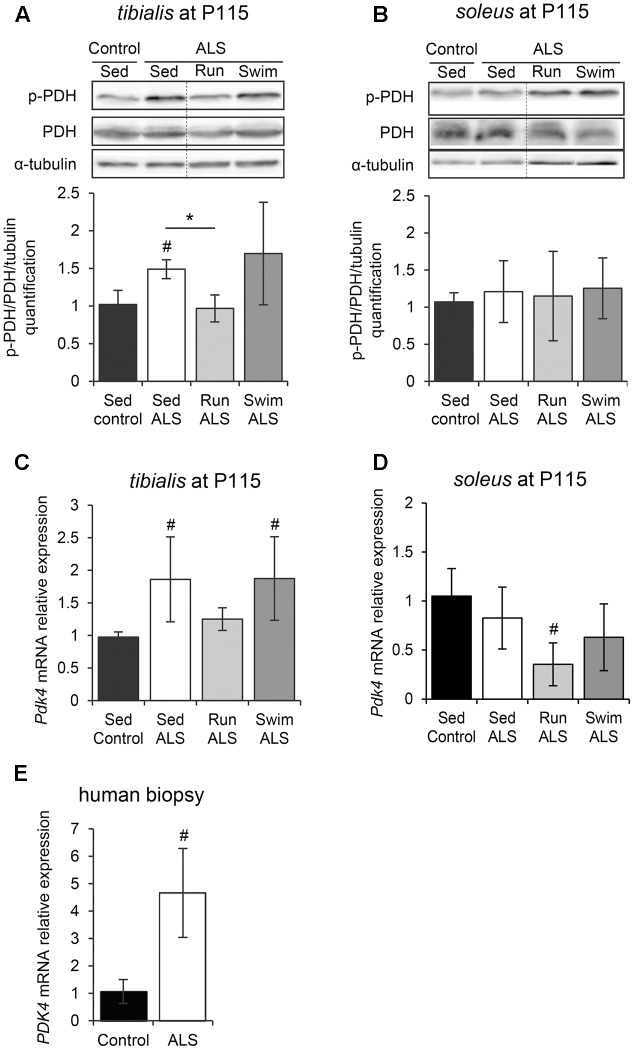
The swimming-based training exerts its beneficial effects independently of PDH modulation. **(A,B)** Western blot analysis (upper panel) and quantification (lower panel) of the phosphorylation status of PDH-E1α in *tibialis* **(A)** and *soleus* **(B)** of Sed Control, Sed ALS, Run ALS, and Swim ALS mice at P115 (*n* = 3). Dotted lines on Western blot images symbolize some removed interspacing lanes for a side-by-side display of samples from all groups. **(C,D)** Quantification by RT-qPCR of *Pdk4* expression levels in *tibialis* **(C)** and *soleus* **(D)** of Sed Control, Sed ALS, Run ALS, and Swim ALS mice at P115 (*n* = 5). mRNA expression levels were normalized with *Rps26* mRNA. **(E)** Quantification by RT-qPCR of *PDK4* mRNA expression levels in human muscular biopsies (control patients *n* = 6; ALS patients *n* = 10). mRNA expression levels were normalized with *RPL13*. All data are shown as mean ± SD; # and ^∗^ indicate significance (with *P* < 0.05) relative to the control and between ALS conditions, respectively.

Pyruvate, a glycolysis by-product, can be metabolized in two different ways in muscles, i.e., the anaerobic pathway that will result in the production of lactate, or the aerobic pathway that will result in the production of acetate. Consequently, we investigated the regulation of the PDH complex, which is pivotal in determining the partitioning of anaerobic compared with aerobic use of pyruvate produced by the glycolysis. The PDH E1-alpha subunit is considered as the on/off switch of the PDH complex, since its phosphorylation by the PDH kinase 4 (PDK4) in the muscle leads to PDH complex inactivation (Wu et al., 1999). Importantly, the alteration of PDK4 expression has been recently highlighted in ALS skeletal muscle (Palamiuc et al., 2015). Therefore, we analyzed the level of PDH E1-alpha phosphorylation in *tibialis* and *soleus* of SOD1(G93A) ALS mice. The level of PDH E1-alpha phosphorylation was significantly higher in the ALS *tibialis* compared to controls (**Figure 4A** and Supplementary Figure S2A), while no significant change was found in the *soleus* (**Figure 4B** and Supplementary Figure S2B). We next evaluated the level of *Pdk4* mRNA expression by RT-qPCR. As previously found in the SOD1(G86R) ALS mouse model (Palamiuc et al., 2015), and consistently with higher PDH phosphorylation levels, ALS *tibialis* displayed significantly higher *Pdk4* expression levels compared to control muscles (**Figure 4C**), whereas no significant change was found in the *soleus* (**Figure 4D**). Interestingly, the effects of physical exercise in ALS *tibialis* altered the PDH phosphorylation status in an exercise-type dependent manner. Indeed, the running-based training induced a significant decrease in PDH E1-alpha phosphorylation and *Pdk4* mRNA expression levels in ALS *tibialis* when compared to sedentary ALS mice (**Figures 4A,C**). By contrast, the swimming-based training had no effect on PDH activation pattern, nor on *Pdk4* mRNA expression levels (**Figures 4A,C**), strongly suggesting that all the swimming-induced changes in ALS *tibialis* unexpectedly occurred independently of PDH modulation. In the *soleus*, only the running-based training induced a significant decrease of *Pdk4* mRNA expression, without a significant change in PDH E1-alpha phosphorylation pattern (**Figures 4B,D**).

We confirmed these findings in human samples from ALS patients. Indeed, we found an increase in *PDK4* mRNA expression ALS when compared to control muscle specimens (**Figure 4E**), as previously reported (Palamiuc et al., 2015).

Taken together, these data indicated that the mechanisms underlying both the ALS-induced alteration of glucose uptake and glycolysis and the beneficial effects of the swimming-based training on GLUT4 and GAPDH expression were independent of a regulation at the PDH level.

### The ALS-Induced Alteration of Autophagic Molecular Component Expression in Skeletal Muscles Is Limited by Swimming Exercise

**FIGURE 5:**
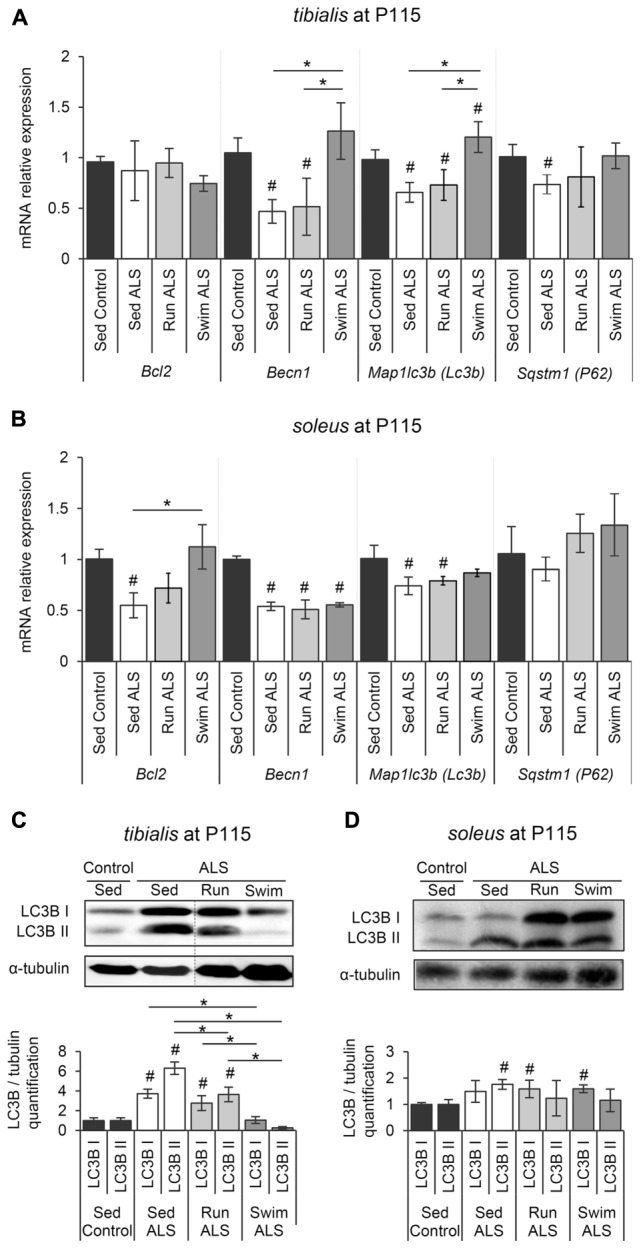
The swimming-based training restores the autophagic flux in ALS skeletal muscles. **(A,B)** Quantification by RT-qPCR of the expression of *Bcl2*, *Becn1*, *Lc3b*, and *P62* mRNA levels in *tibialis* **(A)** and *soleus* **(B)** of Sed Control, Sed ALS, Run ALS, and Swim ALS mice at P115 (*n* = 5). mRNA expression levels were normalized with *Rps26* mRNA. **(C,D)** Western blot analysis (upper panel) and quantification (lower panel) of LC3B I and LC3B II proteins in *tibialis* **(C)** and *soleus* **(D)** of Sed Control, Sed ALS, Run ALS, and Swim ALS mice at P115 (*n* = 3). Dotted lines on Western blot images symbolize some removed interspacing lanes for a side-by-side display of samples from all groups. All data are shown as mean ± SD. # and ^∗^ indicate significance (with *P* < 0.05) relative to the control and between ALS conditions, respectively.

We next investigated whether autophagy, essential for the maintenance of cellular homeostasis and altered in ALS muscles (Dobrowolny et al., 2008; Onesto et al., 2011; Crippa et al., 2013; Xiao et al., 2015), could be involved in the swimming-induced benefits for carbohydrate metabolism in ALS muscles. Among the different systems of autophagy, the Bcl2/Beclin1 (BECN1)-dependent autophagic pathway seems to play an important role in the exercise-induced muscle adaptation of the glucose metabolism in healthy conditions, including GLUT4 relocalization to the sarcolemma and subsequent increase of glucose intake (He et al., 2012). These data prompted us to evaluate the status of this autophagic pathway in *tibialis* and *soleus* of SOD1(G93A) ALS mice. We first evaluated the expression levels of *Bcl2*, *Becn1*, *Map1lc3b (Lc3b)*, and *Sqstm1 (P62)* by RT-qPCR. In sedentary ALS *tibialis*, *Becn1*, *Map1lc3b (Lc3b)*, and *Sqstm1 (P62)* mRNA were significantly down-expressed when compared to controls, whereas no significant change was found for *Bcl2* expression (**Figure 5A**). In the *soleus* of sedentary ALS mice, *Bcl2*, *Becn1*, and *Map1lc3b (Lc3b)* mRNA expressions were down-regulated, with no significant change for *Sqstm1 (P62)* (**Figure 5B**). To complete these results at the protein level, we analyzed LC3B levels by western blot in ALS *tibialis* and *soleus*. Contrasting to the mRNA expression data, we found an accumulation of the two forms of LC3B, LC3B-I and LC3B-II, the lipidated version of LC3B-I involved in autophagosome formation, in ALS *tibialis* when compared to controls (**Figure 5C** and Supplementary Figure S3A). In the ALS *soleus*, we found an accumulation of LC3B-II expression levels, when compared to controls, while no statistical difference was observed for LC3B-I (**Figure 5D** and Supplementary Figure S3B).

When we looked at the ALS *tibialis* after mouse training, we found that, even if both exercise paradigms induced a significant decrease of LC3B-I and LC3B-II proteins compared to sedentary ALS muscles, only the swimming-based training induced a decrease resulting in LC3B protein levels comparable to controls (**Figure 5C**). This decrease at the protein levels was associated with an increase in *Map1lc3b (Lc3b)* mRNA expression levels in ALS *tibialis* (**Figure 5A**). This increase was also found for *Becn1* and *Sqstm1 (P62)* mRNAs, with no effect for running (**Figure 5A**). In the ALS *soleus*, only swimming exercise restored *Bcl2* mRNA expression (**Figure 5B**) while no significant effect was observed at the protein level of LC3B for both exercises (**Figure 5D**).

Taken together, these data strongly suggested that the swimming-based training only improves the expression of the main autophagic molecules in ALS muscles.

### Specific Exercise-Induced GLUT4 and GAPDH Expression in ALS Muscles Is Linked to Autophagy

**FIGURE 6:**
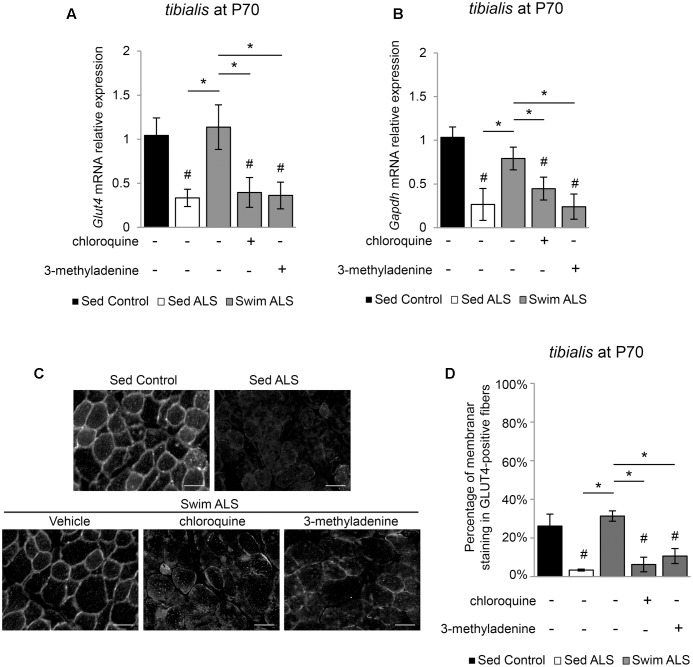
Swimming-benefits on GLUT4 and GAPDH in ALS tibialis is linked to exercise-induced autophagy. **(A,B)** Quantification by RT-qPCR of *Glut4* **(A)** and *Gapdh* **(B)** mRNA expression levels in Sed Control, Sed ALS mice and after 3 days of swimming (Swim ALS) with or without autophagy inhibition by chloroquine (CQ) or 3-methyladenine (3-MA) (*n* = 3). mRNA expression levels were normalized with *Rps26* mRNA. **(C,D)** Immunodetection of GLUT4 in *tibialis* muscle transversal section at P70 (scale bar: 20 μm) **(C)** and analysis of GLUT4 subcellular localization in *tibialis* stained fibers **(D)** in Sed Control, Sed ALS and after 3 days of swimming (Swim ALS mice) with or without autophagy inhibitor with CQ or 3-MA. All data are shown as mean ± SD. # and ^∗^ indicate significance (with *P* < 0.05) relative to the control and between ALS conditions, respectively.

We next investigated whether the swimming-induced enhancement of GLUT4 and GAPDH expression in ALS muscles was dependent on autophagy. We submitted a population of P70 SOD1(G93A) ALS mice to a swimming-based training for 3 consecutive days with or without a blocking of lysosomal degradation by chloroquine (CQ) or the autophagosome formation by 3-methyladenine (3-MA). We observed that, in the early stages of the disease, the expression of *Glut4* and *Gapdh* mRNA was already altered in the *tibialis* of ALS mice and that a 3-days swimming-based training was sufficient to reactivate *Glut4* and *Gapdh* mRNA expression (**Figures 6A,B**). Interestingly, autophagy inhibition with CQ or 3-MA impaired the swimming-induced increase of *Glut4* and *Gapdh* mRNA expression in the *tibialis* of trained ALS mice (**Figures 6A,B**). Like in late stages of the disease, we observed an alteration of GLUT4 subcellular localization in the *tibialis* myofibers of sedentary ALS mice at P70 (**Figures 6C,D**), with a significant decrease of the sarcolemma level localization compared to controls (**Figures 6C,D**). As expected, a 3-day swimming exercise was sufficient to induce a relocalization of GLUT4 to the cell periphery (**Figures 6C,D**). This swimming-induced GLUT4 relocalization was totally abolished by autophagy inhibition whatever the mode of inhibition (CQ or 3-MA) (**Figures 6C,D**).

Taken together, these data suggest that the benefits induced by an anaerobic exercise such as swimming for improving glucose uptake in fast-twitch ALS muscles, are linked to autophagy.

### The Swimming-Based Training Re-equilibrates Gene Expression Related to Fatty Acid Metabolism in the ALS *Tibialis*

We finally asked whether the swimming-induced improvement of glucose metabolism in ALS *tibialis* could limit the lipid hypermetabolism found in ALS muscles (Dupuis et al., 2004b).

**FIGURE 7:**
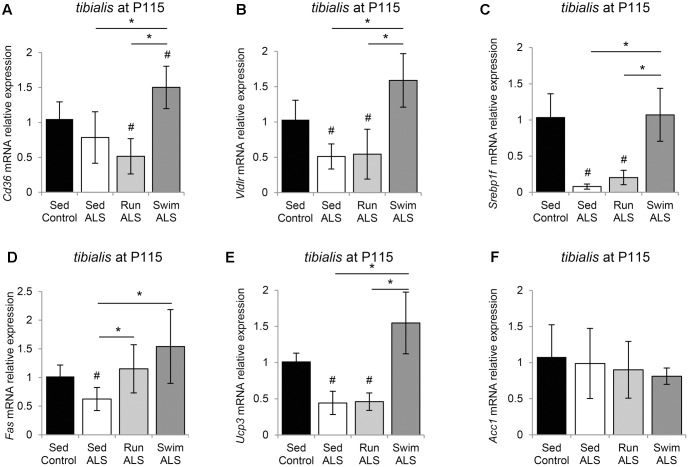
The swimming-based training re-equilibrates gene expression related to fatty acid uptake and lipogenesis in the ALS tibialis. **(A–F)** Quantification by RT-qPCR of gene expression. *Cd36* **(A)**, *Vldlr* **(B)**, *Srebp1c* **(C)**, *Fas* **(D)**, *Ucp3* **(E)**, and *Acc1* **(F)** mRNA expression levels in *tibialis* of Sed Control, Sed ALS, Run ALS, and Swim ALS mice at P115 (*n* = 5). mRNA expression levels were normalized with *Rps26* mRNA. All data are shown as mean ± SD. # and ^∗^ indicate significance (with *P* < 0.05) relative to the control and between ALS conditions, respectively.

We first looked at the expression profile of the two fatty acid transporters CD36 (Cluster of Differentiation 36) and VLDLR (Very Low Density Lipoprotein Receptor) in the *tibialis* of controls and sedentary and trained ALS mice. We found that the levels of *Vldlr* expression were decreased in ALS but no change was observed for *Cd36* expression (**Figures 7A,B**). As expected, the swimming-based training induced a significant increase in *Cd36* and *Vldlr* expression levels, suggesting an improvement in lipid uptake. By contrast, the running-based training had no effect (*Vldlr*) or even induced a significant decrease (*Cd36*) in the expression of these fatty acid transporters (**Figures 7A,B**).

We next looked at the expression levels of several major genes involved in lipogenesis (Liu et al., 2009), including genes coding for the transcription factor *Srebp-1c* (Sterol Regulatory Element-Binding Protein 1c), *Fas* (Fatty Acid Synthase), *Ucp3* (Uncoupling protein 3), and *Acc1* (Acetyl-CoA Carboxylase 1). We found that the expression levels of *Srebp-1c* were profoundly lowered in ALS *tibialis* in comparison to controls (**Figure 7C**). The swimming-based training induced a significant reactivation of *Srebp-1c* expression in ALS *tibialis*, whereas no effects could be recorded with the running-based training (**Figure 7C**). Interestingly, the expression profile of *Fas* (**Figure 7D**) and *Ucp3* (**Figure 7E**) paralleled those of *Srebp-1c*, with a decrease in sedentary ALS *tibialis*, and a significant increase in swimming-based trained muscles. The running-based training had no effect on the expression of *Ucp3* but induced a significant increase in *Fas*. By contrast, *Acc1* expression levels did not display significant alterations in its mRNA expression, in any of the experimental conditions (**Figure 7F**).

Taken together, these data suggest that lipid metabolism is improved by swimming exercise in the ALS *tibialis*.

### The Swimming-Based Training Induced Triglyceride Accumulation in ALS *Tibialis*

**FIGURE 8:**
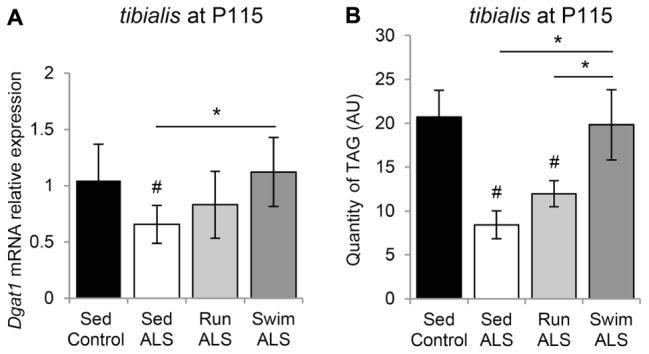
The swimming-based training induced triglyceride accumulation in ALS tibialis. **(A)** Quantification by RT-qPCR *Dgat1* mRNA expression levels in *tibialis* of Sed Control, Sed ALS, Run ALS, and Swim ALS mice at P115 (*n* = 5). mRNA expression levels were normalized with *Rps26* mRNA. **(B)** HPLC analysis and quantification of TAG (triacylglycerol) in *tibialis* of Sed Control, Sed ALS, Run ALS, and Swim ALS mice at P115 (*n* = 5). All data are shown as mean ± SD. # and ^∗^ indicate significance (with *P* < 0.05) relative to the control and between ALS conditions, respectively.

These results, indicating that the expression of several lipogenic genes, i.e., *Fas* and *Ucp3* (Liu et al., 2009) are enhanced by the swimming-based training, prompted us to evaluate the expression profile of diacylglycerol acyltransferase 1 (*Dgat1*), which increases muscle fat content, associated with PDH inhibition and *Ucp3* overexpression (Liu et al., 2009). We found that the levels of *Dgat1* mRNA expression were significantly lower in ALS *tibialis*, when compared to controls (**Figure 8A**). Importantly, the swimming-based training induced a significant increase in *Dgat1* expression levels (**Figure 8A**) compared to sedentary ALS, suggesting that, in addition to favoring the uptake of fatty acids, the swimming-based training might favor the neo-synthesis of lipids. Compared to sedentary ALS, a significant increase in *Dgat1* expression was also recorded in the running *tibialis*, albeit lower than in swimming (**Figure 8A**).

Finally, we quantified by HPLC the levels of triacylglyceride (TAG) in the sedentary and trained ALS *tibialis* and control muscles. As expected, we found a decrease in TAG quantity in the ALS *tibialis* when compared to controls (**Figure 8B**). Importantly, the swimming-based training induced a 2-fold increase in TAG levels in the ALS *tibialis*, reaching levels comparable to controls, whereas the running-based training only increased TAG levels by 1.4-fold (**Figure 8B**).

Taken together these data suggested that swimming could favor fat deposition in ALS muscle.

## Discussion

Here, we report the first lines of evidence indicating that a specific exercise paradigm, based on a high intensity and amplitude swimming exercise, significantly improves glucose metabolism in ALS mice. These swimming-induced benefits were associated with changes in skeletal muscle energetic metabolism of ALS mice, leading to energetic fuel shifts toward glucose re-use and fat deposition. Importantly, the beneficial effects of the swimming-based training occurred independently of PDH modulation, providing a promising way to synergize the benefits induced by PDK4 targeting recently shown to be efficient in reducing metabolic alterations in ALS muscles (Palamiuc et al., 2015).

In the present study, we demonstrated that a significant glucose intolerance occurs in SOD1(G93A) ALS mice since the presymptomatic phase of the disease (P70). ALS-induced glucose intolerance, albeit well established in human ALS patients (Poulton and Rossi, 1993; Pradat et al., 2010), was not found in previous studies using male SOD1(G86R) ALS mice, in which an increase in glucose tolerance was revealed (Dupuis et al., 2004b) or in a mix of male and female SOD1(G93A) ALS mice, in which no alteration of glucose tolerance could be recorded (Smittkamp et al., 2014). The latter discrepancy is likely to reflect the difference in disease severity, genetic background or sex bias (James and Walker, 1985; Mu et al., 1999; Dupuis et al., 2004a; Deforges et al., 2009; Shimizu et al., 2012). In addition, our data are strengthened by a metabolomic approach using NMR, performed for the first time on ALS mouse muscles, that showed a significant decrease in glucose concentration in the SOD1(G93A) ALS *tibialis*, which is expected, from a fast-twitch muscle, to preferentially use glucose for its energetic metabolism. Although glucose concentration levels have not been investigated in muscles of SOD1(G86R) ALS mice, an excess of glycogen associated with an inhibited glycolysis has been found in the *tibialis* of these mice (Palamiuc et al., 2015), suggesting a defect in glucose availability for ALS muscle, even in a context of an excessive glucose uptake (Fergani et al., 2007). Taken together, these data point toward a defect in free glucose availability for fueling glycolysis in fast-twitch ALS muscles.

Most importantly, the swimming-based training significantly improved glucose tolerance in SOD1(G93A) ALS mice (**Figure 1**). The running-based training had much more modest effects. In the specific context of ALS, characterized by an excessive lipidic catabolism (Dupuis et al., 2004b) that is necessarily oxidative, a running-induced attenuation of the glycolytic energetic pathway is likely to reinforce the oxidative metabolism (Romijn et al., 1993; van Loon et al., 2001), which could further promote the use of lipids as energetic substrate. By contrast, favoring a metabolic shift toward a more glycolytic muscular metabolism, using high intensity exercise types including swimming, is likely to improve glucose tolerance and glucose use by the solicited muscles. The present results contrast with previous studies that have shown that high intensity running-based protocols are detrimental to ALS mice (Mahoney et al., 2004; Carreras et al., 2010). One possible explanation for this discrepancy is that, even at a relatively high intensity, a running-based training in mice would unlikely provide the necessary workload to promote the activation of the glycolytic pathways. The swimming-based training we used was shown to induce a lactate production and specific muscular adaptations (Grondard et al., 2008), including fast-twitch fiber transitions, as classically found in high intensity exercises (Grondard et al., 2008). Therefore, further work would be useful to record the evolution of metabolic indices and analyze skeletal muscle phenotype adaptations, notably the typology, in high intensity running-based training protocols to verify the workload impact on the metabolic pathways.

The important energetic needs of skeletal muscles make it one of the most voracious tissues in energetic substrates. The pumping of circulating glucose by the myofibers is regulated by insulin-elicited signals that lead to the translocation of GLUT4 glucose transporter from intracellular storage micro-vesicles to the sarcolemma. This feature makes GLUT4 a key player in normal glucose homeostasis but also an important factor in insulin resistance (Ren et al., 1995; Leturque et al., 1996; Tsao et al., 1996) and glucose intolerance, two hallmarks of ALS (Reyes et al., 1984; Pradat et al., 2010; Dupuis et al., 2011; Sacca et al., 2012). One important finding from our study is that the decrease in GLUT4 expression levels, found in human and mouse ALS muscles, is associated, in mice, with a decrease expression at the cell periphery (**Figure 2**). Both defects would explain glucose uptake inhibition by ALS muscles. In addition, we, and others, have found impairments in GAPDH expression levels in ALS muscles (**Figure 3**; Calvo et al., 2008), which were associated with a reduced GAPDH enzymatic activity (Pierce et al., 2008). This could be a consequence of glucose uptake defects that are likely to impact downstream genes in glucose metabolism pathways. Accordingly, we report here an alteration of PDH activation pattern associated with *Pdk4* over-expression in the *tibialis* of SOD1(G93A) mice as recently evidenced in SOD1(G86R) mice (Palamiuc et al., 2015). Interestingly, the swimming-based training restored muscular GLUT4 expression, and increased GAPDH expression, but was unable to restore PDH activation pattern, as shown by the persistently high levels of phospho-PDH and of *Pdk4* expression (**Figure 4**). Accordingly, blood lactate levels were significantly increased by the swimming-based training, strongly suggesting that the produced pyruvate is used, a least in part, to enhance the anaerobic glycolytic pathway in ALS muscles.

It is astonishing to note that the swimming-induced shift to anaerobic glycolytic pathway is associated with an enhanced fat storage in ALS *tibialis*, likely resulting from (1) lipid uptake, as suggested by the increase of *Vldlr* and *Cd36* expression levels, and (2) TAG synthesis, as suggested by the swimming-induced expression of lipogenesis genes such as *Fas*, *Acc1*, and *Dgat1*. Interestingly, in humans, the exercise-induced accumulation of intramuscular TAG and protection against insulin resistance correlated with an increase in DGAT1 expression levels (Schenk and Horowitz, 2007). Moreover, the skeletal muscles of mice overexpressing DGAT1 display an increase of muscular triglyceride synthesis coupled with fatty acid oxidation (Liu et al., 2009). In addition, and in line with our results in the swimming-trained ALS *tibialis*, *Glut4, Cd36, Pdk4*, and *Ucp3* expression levels were increased in DGAT1-overexpressing muscles (Liu et al., 2009).

**FIGURE 9:**
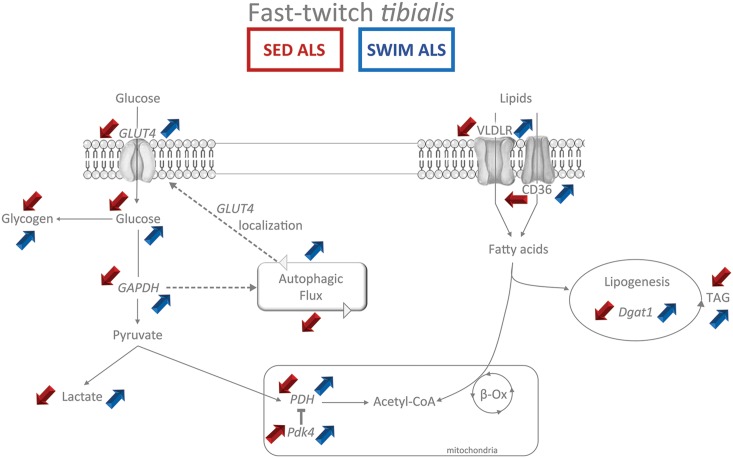
Synthetic summary of energetic metabolism status in fast-twitch tibialis of sedentary and swimming-trained ALS mice at P115. The main experimental alterations observed in the glycolytic and lipidic metabolism (Red arrows) in the fast-twitch tibialis of sedentary ALS (SED ALS) compared to sedentary controls are contrasting with the beneficial effects (blue arrows) of the swimming-based training in ALS mice (SWIM ALS) at 115 days of age. The main metabolites appear in normal typeface while enzymes appear in italic typeface. The colored up arrows correspond to an increase while the down arrows correspond to a decrease.

Moreover, we report in the present study that the swimming-induced improvement in carbohydrate metabolism in the ALS *tibialis* is linked to the autophagy improvement in fast-twitch ALS muscle. Alterations in the expression of autophagy markers have been already reported in the muscles of ALS mice, albeit with variable and even contradictory results probably reflecting differences in disease severity and in muscle types (Dobrowolny et al., 2008; Onesto et al., 2011; Crippa et al., 2013; Olivan et al., 2015). Yet, while the increase in LC3B and P62 expression levels in quadriceps muscles (Crippa et al., 2013) or in unseparated hindlimb ALS muscle tissue (Olivan et al., 2015) was interpreted as an enhancement of the autophagic flux in end stage ALS muscles, the impairment of autophagosome formation in live ALS myofibers was interpreted as a suppression of the autophagic pathway (Xiao et al., 2015). Here, we compared the expression of autophagy markers in two muscles that are known to be differentially affected by the disease, i.e., the relatively spared *soleus* vs. the highly affected *tibialis*. We found a dramatic accumulation (more than fivefold) of the main autophagy protein LC3B, whatever the form I or II, in the ALS *tibialis* whereas this accumulation was less than twofold in the ALS *soleus*, and only concerned LC3B II. Interestingly, LC3B protein accumulation correlated with a significant decrease in *Map1lc3b (Lc3b)* mRNA levels, suggesting a negative feedback on gene transcription due to protein accumulation. Taken together with previous observations (Xiao et al., 2015), these data substantiate the hypothesis of an autophagic flux blockade in ALS muscles. Since the saturation of the autophagic flux due to aggregation of the mutated SOD1 protein is unexpected in muscles (Wei et al., 2012), the reason for the autophagic protein expression alterations could be a consequence of the ALS-induced defective energetic metabolism. Yet, the flux of intracellular vesicles, including autophagosome, mainly depends on anaerobic glycolysis that occurs directly at the level of the moving vesicles (Zala et al., 2013). The glucose intolerance found in ALS would compromise the whole vesicular traffic in muscle, leading both to a reduction in the autophagic flux, and in turn to an even more important decrease in GLUT4 concentration at the plasma membrane, progressively worsening muscular metabolic impairments. In this context, the strong glucose demand induced by high intensity swimming exercise in the fast-twitch glycolytic *tibialis* muscle, would force glucose uptake to the exercised muscle, feeding the glycolytic pathway, mobilizing the vesicular flux leading to GLUT4 mobilization to the cell membrane, and ultimately resulting in an improvement in muscular energetic metabolism. Accordingly, we report here that autophagy inhibition leads to a significant reduction in the swimming-induced increase of GLUT4 and GAPDH expression levels in the ALS *tibialis* (**Figure 6**). However, the exact mechanism(s) triggered by swimming exercise and resulting in the improvement of both autophagy and GLUT4 expression still remains to be identified (**Figure 9**).

One important question raised by our results is the potential impact of muscle metabolic alterations, including autophagy impairments, on ALS motor neuron survival. An altered muscle metabolism might destabilize neuromuscular junctions, ultimately resulting in muscle denervation and motor neuron death (Dupuis et al., 2009). Accordingly, the present data show that *tibialis*, which is mostly innervated by ALS-sensitive fast motor neurons, is more severely affected by metabolic alterations compared to the *soleus*, innervated by ALS-spared slow motor neurons. Moreover, the swimming-induced metabolic shift to the glycolytic pathway, notably in the fast-twitch *tibialis*, correlated with a significant neuroprotection at the level of the fast motor neurons in the mouse ALS spinal cord (Deforges et al., 2009), suggesting that muscle metabolism status may impact motor neuron survival. The running-based training induced no (Veldink et al., 2003; Deforges et al., 2009), transient (Carreras et al., 2010) or weak and sustained (Kaspar et al., 2005) neuroprotection. As expected for a lower intensity exercise, the running would favor the oxidative metabolism in solicited muscles. Likewise, reinforcing the oxidative pathway by a running-based training is seemingly contributing to alter energetic metabolism in ALS muscle and ultimately favor the neurodegenerative process. Thus, the present data suggest that, in addition to PDK4 inhibition (Palamiuc et al., 2015), the switching to glycolytic muscle metabolism, induced by well-designed exercise programs, may provide synergistic beneficial effects for ALS patients.

## Author Contributions

CD and SD made the majority of the cellular and molecular experiments and analyzed the data. CD contributed to the writing of the manuscript. AL performed the lipidomic analysis. CCa and GBe performed the RMN analysis. GBr and FS provided the human muscle samples, contributed to data collection (human samples) and analyzed the data. JB and FD performed the enzyme analysis and contributed to the editing of the manuscript. OB, LW, LH, and DD’ performed the analysis of autophagy inhibition, analyzed the data and OB, LW, and LH contributed to the writing of the manuscript. CCh and CM contributed to the analysis of the energetic data and to the editing of the manuscript. FC contributed to data collection, analyzed data and wrote the manuscript. All authors contributed to the design and completion of the experiments.

## Conflict of Interest Statement

The authors declare that the research was conducted in the absence of any commercial or financial relationships that could be construed as a potential conflict of interest.
