# Supplementary figures and images for: Specific Physical Exercise Improves Energetic Metabolism in the Skeletal Muscle of Amyotrophic-Lateral- Sclerosis Mice
Source: Front Mol Neurosci. 2017 Oct 20;10:332. doi: 10.3389/fnmol.2017.00332 (PMC5655117; doi:10.3389/fnmol.2017.00332)

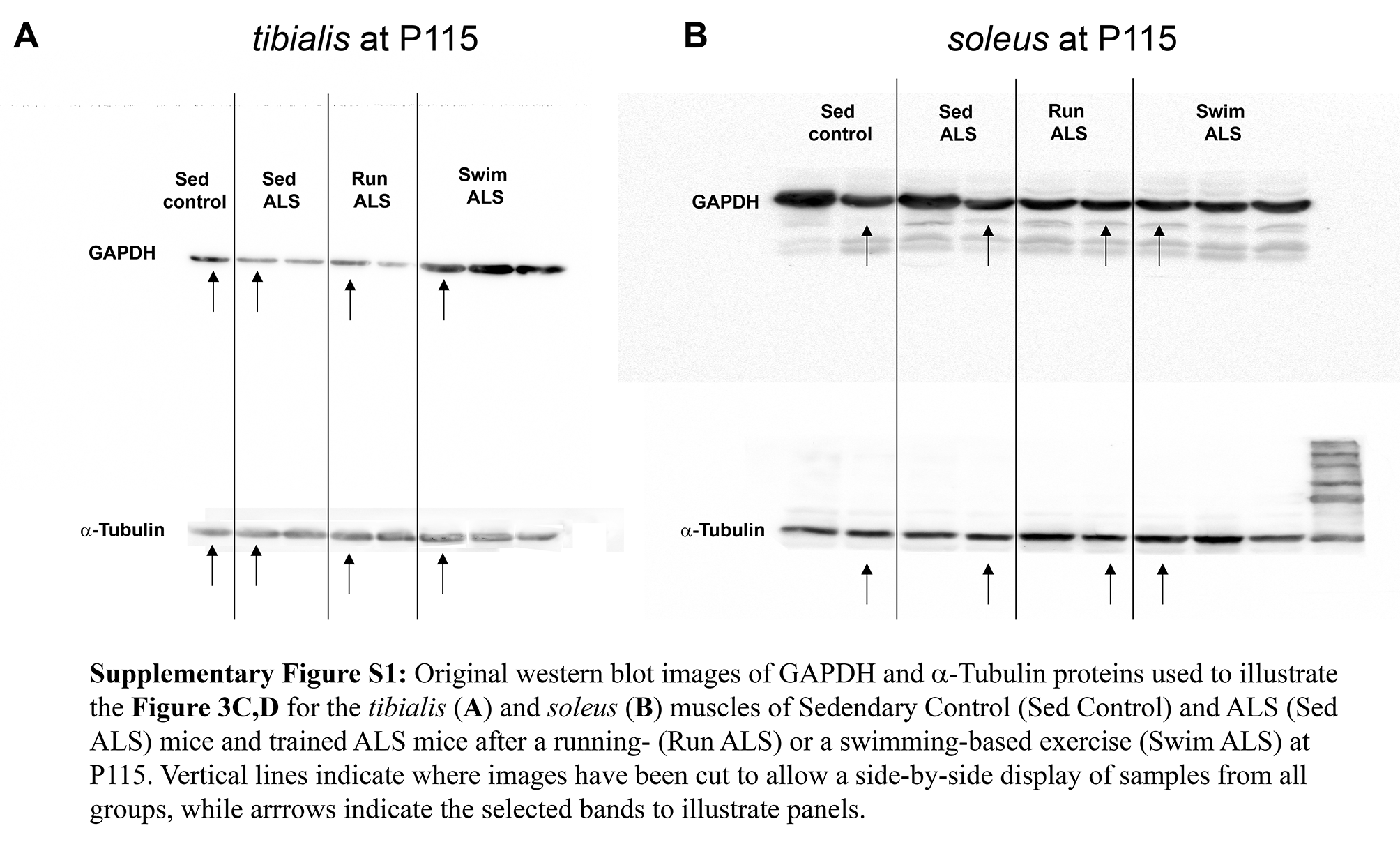

Supplement: Supplementary file 1 [file Image_1.tif]

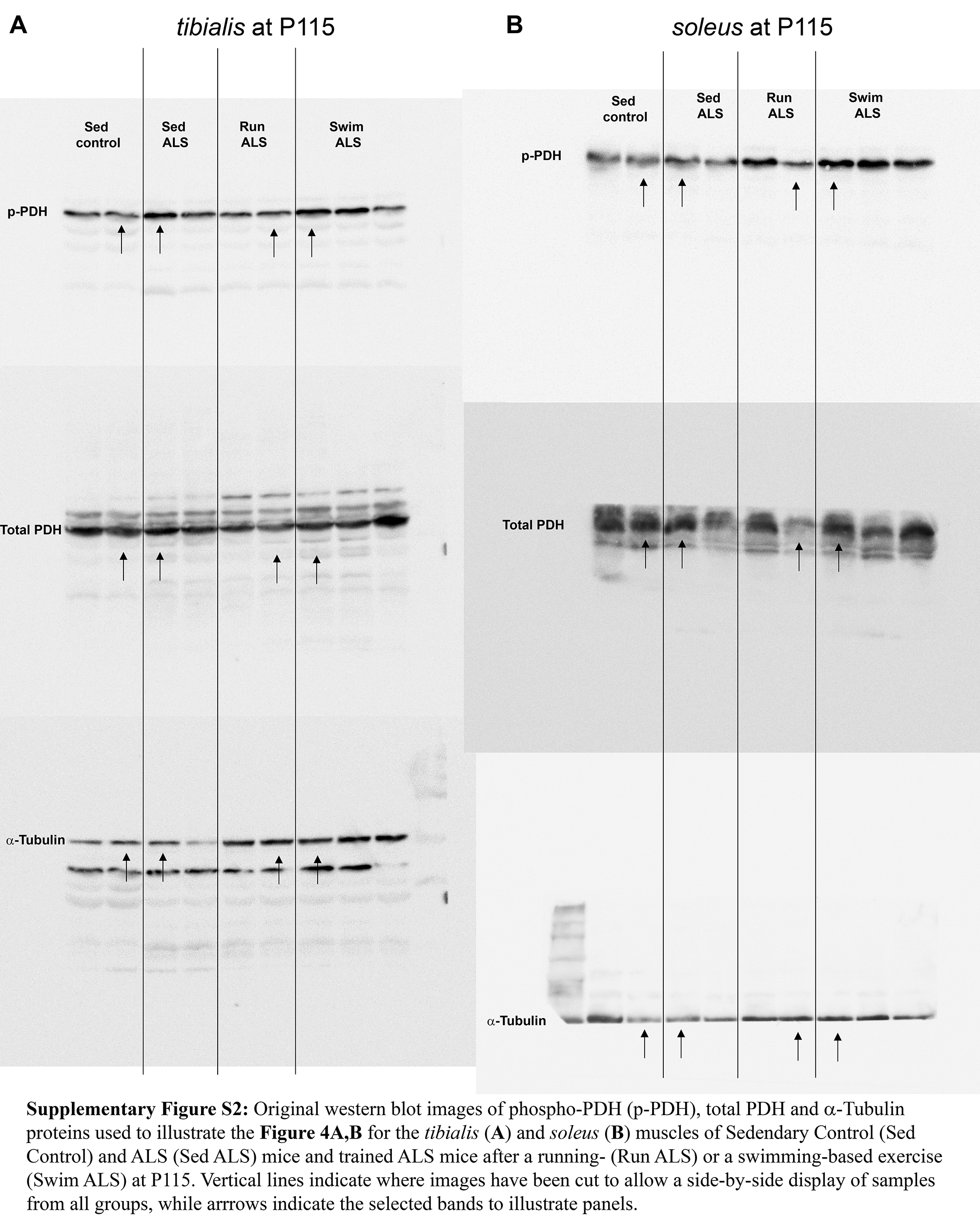

Supplement: Supplementary file 2 [file Image_2.tif]

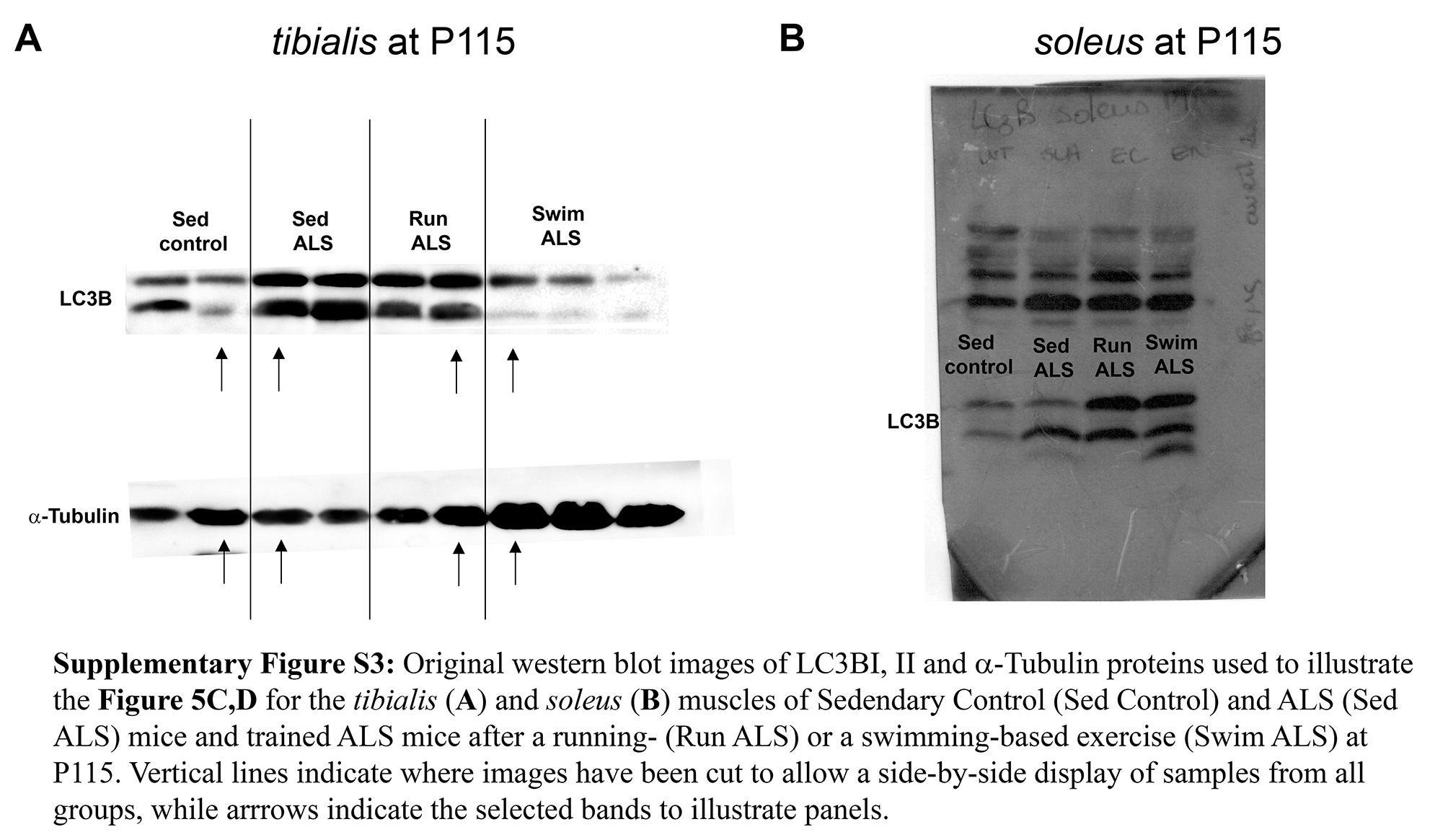

Supplement: Supplementary file 3 [file Image_3.tif]
